# Impact of hybrid solar and wind distributed generators on transient stability of captive power systems

**DOI:** 10.1038/s41598-026-51606-8

**Published:** 2026-05-22

**Authors:** P. S D Bhimaraju, K V S Ramachandra Murthy, Kollu Ravindra

**Affiliations:** 1 JNTUK, Kakinada, Andhra Pradesh India; 2 Aditya University, Surampalem, Andhra Pradesh India; 3 University College of Engineering Kakinada, JNTUK, Kakinada, Andhra Pradesh India

**Keywords:** Transient stability analysis, Industrial power systems, Solar and Wind Distributed Generator Units (Solar and Wind DGs), Critical Clearing Time (CCT), Renewable energy integration, Energy science and technology, Engineering

## Abstract

This paper investigates the impact of solar and wind distributed generators on the transient stability of a 39-bus industrial power system. The system is analyzed under different configurations, including wind-only, solar-only, and hybrid DG integration. Critical Clearing Time (CCT), active power, and reactive power are used as performance metrics. The results show that DG integration improves system stability compared to the baseline case. Specifically, the CCT increases from 0.018 s (without DG) to 0.0248 s with wind DG, and up to 0.3168 s with solar DG. The hybrid configuration results in a CCT of 0.0297 s. These results indicate that solar DG provides the highest improvement in transient stability due to better voltage support characteristics. The study highlights the importance of DG type and placement in improving stability and provides insights for industrial power system design.

## Introduction

Transient stability refers to a power system’s ability to maintain stability amidst rapid load variations^1^. It addresses electro-mechanical instabilities and is determined by analyzing internal machine angle variation. A fault is considered transiently stable if it manages rotor angle oscillations and stabilizes within a safe operational range^2^. As environmental degradation and fossil fuel consumption decrease, there is a growing interest in clean energy sources like fuel cells and spinning turbines. Conventional power systems use centralized generators with synchronous operation, but the power system is moving towards distributed power generation, integrating renewable energy on a larger scale^3^. Distributed energy production can be defined as a small-scale power plant that operates with the help of combustion as well as non-combustion technologies. Distributed power generation (DG) is gaining importance in the generation of electric energy^4^.

RES can minimize emissions, cut down construction and operating costs, and also enhance feeder voltage characteristics^5^. Distributed generation (DG) systems like solar and wind are advantageous over traditional power systems in several ways^6^. The integration of DG can result in the maximum outcome if the resource is allocated appropriately, but if not, it will threaten security and stability. DC integration is far superior in the control of hybrid systems and scales solar more effectively. These include DG technologies such as biomass, solar, and wind generators that come in sizes of a few kW up to a few MW^7^. Capacitor banks are applied to eliminate output fluctuations and avoid grid power flow interchange. Standalone generating stations can enhance reliability and efficiency. The increasing energy demand and installed capacity of DG are compelling power producers to look for ways to improve other technical characteristics, such as voltage stability and power losses^8^. Generation firms are also trying to push up the penetration of DG at the same time.

DG management techniques are important for controlling numerous micro-sources and sources of energy, including wind and solar power plants^9^. These sources connect to the electrical grid through electronic inverters to maintain the frequency. Nonetheless, temperature and irradiance can affect solar production, thereby influencing the performance of DG^10^. Induction machines are used in wind turbines that convert energy, but many operational problems will emerge as wind energy increases^11^. These issues must be understood by engineers to be able to guarantee the security of the electrical system. Virtual Synchronous Generators (VSG) can enhance the stability of the frequency but at an extra cost as compared to when DGs are connected directly to the grid^12^. All in all, it can be stated that the proper design of DG systems is critical to obtaining the desired performance and safety levels.

To improve system dependability, the study will evaluate how integrating dispersed solar and wind generators affects transient stability in industrial power systems. It will also offer insights and suggestions.

The main contributions of this work are:

- Analysis of transient stability in an industrial power system with solar and wind DG integration.
- Comparison of different DG configurations using Critical Clearing Time.
- Study of the relationship between DG placement and power flow changes.
- Development of a MATLAB/Simulink-based simulation framework for stability evaluation.

The format of the paper is as follows: In section II, prior research on the integration of RES and transient stability analysis is reviewed. The integration of distributed solar and wind generators is provided in section III. The study’s performance measures and simulation are covered in section IV. The paper’s conclusion and some directions for future research are provided in section V.

### Related research

Based on hydroelectric, wind, solar, and thermodynamic power sources, the investigation^13^ used Matlab/Simulink to model Madeira Island’s power-generating mix. The present, the future, and the scenario, including a system for storing energy in batteries, were all considered. According to the findings, in the absence of a battery energy storage system (BESS), the thermal electrical generator desynchronized, resulting in the collapse of the system. Yet, the inclusion of a BESS increased system efficiency and improved the stability of frequencies.

In addition to analyzing government actions, tender activity, obstacles, and prospects, research^14^ looked at India’s energy storage requirements. It determined the financial feasibility of solar power systems with battery storage through a thorough techno-economic analysis. The results add to the conversation about storage options and RES integration in India.

The influence of unexpected variations in solar energy on a commercial transmission system linked to cogeneration facilities was examined in the article^15^. It employed real-time field data along with models to evaluate power quality, performance, and load management. The document also included instructions for additional integration research, as well as recommendations for industry and politicians seeking solar energy absorption.

The employing of utility-run roof solar panels with BESS for improved energy storage and grid resilience was investigated in the research^16^. It considered reverse flow, thermal loading, and voltage increase while analyzing consumer data from eight Indian industries. The research indicated that optimizing peak load, decreasing the usage of diesel generators, and raising the percentage of solar energy might yield the greatest economic advantages. Grid stability, adaptability, and dependability all depended on BESS.

The best places for micro-grids to operate in both normal and extreme weather scenarios were analyzed in the paper^17^. It suggested an all-encompassing approach to deal with those problems. The assessment also emphasized how ESUs could help with resistance and resilience in the future, which would increase the overall system’s dependability and resilience.

By looking at mitigation strategies from current academic papers and technical reports, the study^18^ sought to assess the effects of massive amounts of adoption of RES. The objective was to pinpoint the primary hazards that impact the stability margin of the network, enabling a justifiable augmentation in such margin. Suitable mitigating techniques would be chosen based on the findings.

With an emphasis on cost and emission efficiency, paper^19^ presented an approach for mixed adoption and RES inclusion. The model, which attempted to achieve significant decreases in emissions of gases, was contrasted with hybrid and connected to the grid models. The study examined the Ramchandrapura feeder test feeder with actual spatial data and Homer-Pro programs, considering the ecological effects of Distributed Energy Resources (DER) inclusion.

The study demonstrated that the integration of Nuclear Power Plants (NPP) cooling towers with Solar Chimney Power Plants (SCPP) has been advanced in the production of electricity and water^20^. Raising the temperature of the air within the NPP via the excess hot air from the SCPP + NPP system and enhancing the air volume in the chimney depending on time yielded dependable and constant power generation within the SCPP.

A study^21^ proposed a new instantaneous current reference method for a Hybrid Power System (HPS) that seems both autonomous and grid-connected. Composed of battery systems, wind turbine generators, and solar panels, among others the grid-integrated HPS (GI-HPS). The load requirements have been met by controlling the electricity from the hybrid power source and storage components. The suggested energy management system (EMS) has been used for improving the dynamism efficiency and effectiveness of HPS that operate on RES.

RES was explored extensively in the context of the Beijing-Tianjin-Hebei (BTH) Region in a study carried out for the research^22^ to identify the challenges of RES and establish a level playing field of analysis for evaluating the economic and technical prospects of resource flexibility. Besides, the study provided policy recommendations for enhancing flexibility in the area as well as analyzed moderately/ permanently viable ways to enhance flexibility.

Study^23^ evaluated several Water Heat Storage (WHS) projects that analyzed the design process, including the optimal location for placing the solar and wind panels and also the power evacuation arrangement. Besides presenting the identified risks and the corresponding risk management measures, the research also explored regulatory actions that would help enhance WSH in the mix of electricity generation, small irrigation scheme design, and the choice of solar and wind power systems.

With an emphasis on technical, infrastructure, and user factors, research^24^ investigated the difficulties of integrating RES on a wide scale in India. To grasp the intricacies, it drew on additional research and supplier experiences. A sustainable energy future for each sector was highlighted in that report, which focused on system-strengthening measures and addressed reliability, consistency, and grid balance.

Using a bibliometric method, the study^25–30^ employed data mining to find patterns and trends in DG. In addition, current developments were highlighted. By embracing new technology and environmental concerns, the research expanded on previous system performance and optimization. The technique functioned as a guide for DG integration research in future years and may be extended to other study areas.

## Methodology

### System modeling

This section includes the following topics: load modeling, generator modeling, and general power system structure.

### The general structure of the power system

**Fig. 1:**
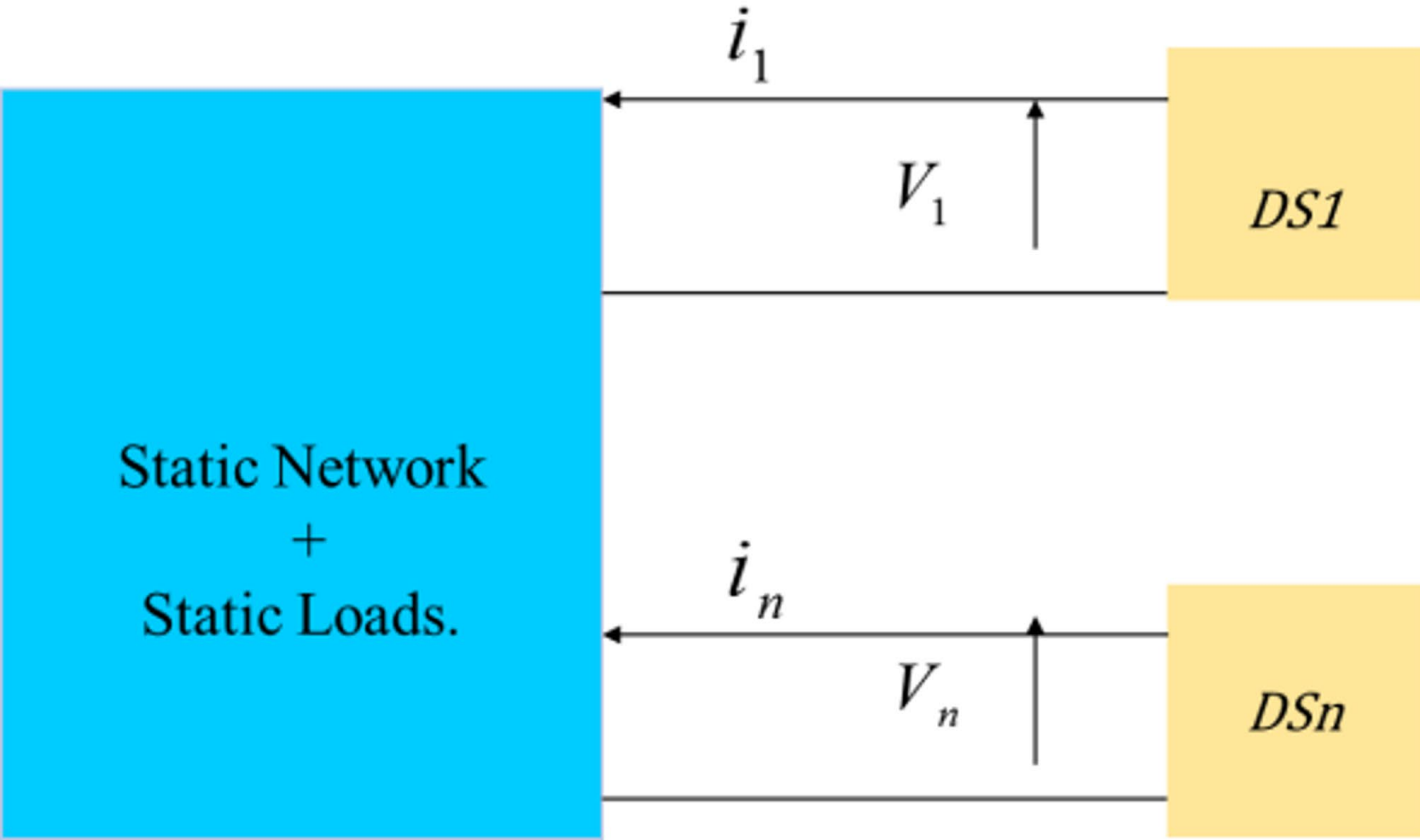
Power System Representation.

Transistor dynamics, reaction (series and shunt), and transmission paths all have comparatively quicker dynamics. In the bus susceptibility matrix, the data transfer system is shown. The electricity system structure is shown in Fig. 1. The files used in the simulation study are network data as an executable file. The transient models of each of the program’s 10 sources are shown on the immediate and lateral axes. The general utilities at Bus No. 2 are modeled as a single source with high inertia.

#### Generator equations

The two ($\:c$ and $\:r$ axis) that correspond to the 3-phase winding are the stator’s representation. Given are the flux linkages related to the winding of $\:the\:c$ and $\:r$ axes.

1 $$\:-\frac{1}{{\omega\:}_{A}}{\dot{\varPsi\:}}_{c}-\left(1+{t}_{h}\right){\varPsi\:}_{r}-{Q}_{b}{j}_{c}={u}_{c}$$

2 $$\:-\frac{1}{{\omega\:}_{A}}{\dot{\varPsi\:}}_{r}-\left(1+{t}_{h}\right){\varPsi\:}_{c}-{Q}_{b}{j}_{r}={u}_{r}$$

Where, $$\:{t}_{h}=\frac{{\omega\:}_{A}-{x}_{q}}{{x}_{A}}$$

3 $$\:{j}_{r}=\frac{{\varPsi\:}_{c}-{{F}^{{\prime\:}}}_{r}}{{w}_{c}^{{\prime\:}}}$$

4 $$\:{j}_{r}=\frac{{\varPsi\:}_{r}-{{F}^{{\prime\:}}}_{c}}{{w}_{r}^{{\prime\:}}}$$

When examining electromechanical dynamics, stator transients are frequently disregarded because of their rapidity. The outcome is the following two output algebraic equations:

5 $$\:-{\varPsi\:}_{r}-{Q}_{b}{j}_{c}={u}_{c}$$

6 $$\:-{\varPsi\:}_{c}-{Q}_{b}{j}_{r}={u}_{r}$$

It is presumed that there are no zero sequence currents present in the stator. Here,$\:{\omega\:}_{A}$ stands for the generator slip, $\:{t}_{H}$ is the base frequency of angular motion in rad/s, $\:{j}_{c},\:and\:{j}_{r}$ are the framework current’s $\:c$ and $\:r$ axis components, and $\:{u}_{c}\:and\:{u}_{r}$ are the machine terminal voltage’s $\:c$ and $\:r$ axis components. Using the transformation, $\:{u}_{hc}\:and\:{u}_{hr}$ are converted to Kron’s comparison frame (D-Q axes) to share an identical axis reference point with the network.

7 $$\:\left[{u}_{hd}\:{u}_{hq}\:\right]=\left[cos\delta\:\:sin\delta\:\:\:-sin\delta\:\:cos\delta\:\:\:\right]\left[{u}_{gD}\:{u}_{gQ}\:\right]$$

The equipment’s terminal voltages’ $\:C$ and $\:R$ axis constituents are represented, respectively, by the parameters $\:{u}_{hC}$and$\:{u}_{hR}$. The angle produced by the $\:C-R$ axis and the $\:c-r$ axes of the synchronously rotating frame is represented by the angle δ. All of$\:{\:j}_{C}$, $\:{j}_{R}$,$\:c$, and $\:r$ are related.Damper winding equation,$\:\frac{d{{E}_{c}}^{{\prime\:}}}{dt}=\frac{1}{{S}_{qo}^{{\prime\:}}}\left[-{F}_{c}^{{\prime\:}}-\left({w}_{r}-{w}_{r}^{{\prime\:}}\right){j}_{r}\right]$ (8)

Field winding equation,$\:\frac{d{{E}_{r}}^{{\prime\:}}}{dt}=\frac{1}{{S}_{qo}^{{\prime\:}}}\left[-{F}_{r}^{{\prime\:}}+{F}_{fd}-\left({w}_{c}-{w}_{c}^{{\prime\:}}\right){j}_{r}\right]$(9).

In this equation, $\:{S}_{q0}^{{\prime\:}}\:and\:\:{S}_{q0}^{{\prime\:}}$ are the transient time constants for the open circuit $\:c$ and $\:r$ axis, respectively, while $\:{F}_{fd}$ represents the field voltage. The relationship between $\:{F}_{c}^{{\prime\:}},\:{F}_{r}^{{\prime\:}},\:{u}_{c},\:and\:{u}_{r}$ is provided by:

10 $$\:{F}_{r}^{{\prime\:}}+{j}_{c}{w}_{c}^{{\prime\:}}={u}_{r}$$

11 $$\:{F}_{c}^{{\prime\:}}+{j}_{r}{w}_{r}^{{\prime\:}}={u}_{c}$$

The generator’s electrical torque can be calculated using the swing Eq. 

12 $$\:{S}_{f}=\frac{{w}_{c}^{{\prime\:}}-{w}_{r}^{{\prime\:}}}{{w}_{c}^{{\prime\:}}{w}_{r}^{{\prime\:}}}{\varPsi\:}_{c}{\varPsi\:}_{r}+\frac{{F}_{c}^{{\prime\:}}{\varPsi\:}_{c}}{{w}_{r}^{{\prime\:}}}+\frac{{F}_{r}^{{\prime\:}}{\varPsi\:}_{r}}{{w}_{c}^{{\prime\:}}}$$

It can be expressed using Eqs. (5) and (6) as:

13 $$\:{\varPsi\:}_{c}={F}_{r}^{{\prime\:}}+{j}_{c}{w}_{c}^{{\prime\:}}$$

14 $$\:{\varPsi\:}_{r}={F}_{c}^{{\prime\:}}+{j}_{r}{w}_{r}^{{\prime\:}}$$

Equation (12) can be combined with Eqs. (13) and (14) to get the following Eq. 

15 $$\:{S}_{f}={j}_{r}{F}_{r}^{{\prime\:}}+{j}_{c}{F}_{c}^{{\prime\:}}+{j}_{r}{j}_{c}\left({w}_{c}^{{\prime\:}}-{w}_{r}^{{\prime\:}}\right)$$

The swing equation can be found using,

16 $$\:t=\frac{1}{{2G}_{h}}\left({S}_{n}-{S}_{f}-{Ds}_{h}\right)$$

17 $$\:t\frac{d\delta\:}{dt}={\omega\:}_{a}\left(slip\right)={\omega\:}_{a}\left({t}_{h}\right)$$

In this equation, $\:{U}_{h}$ represents the machine terminal voltage and $\:{U}_{t}$ represents the (not used in this study) Power System Stabilizer (PSS) output. The following equation describes the condition of the excitation system:

18 $$\:{F}_{fd}=-\frac{1}{{S}_{F}}{F}_{fd}+\frac{{L}_{F}}{{S}_{F}}\left({U}_{ref}+{U}_{t}-{U}_{h}\right)$$

19 $$\:{U}_{h}=\sqrt{\left({U}_{gD}^{2}+{U}_{gQ}^{2}\right)}$$

#### Connecting generator to the network

The relationship between $\:{F}_{r}^{{\prime\:}},\:\:{F}_{c}^{{\prime\:}}\:and\:\:{u}_{c},\:\:{u}_{r}$is provided by,

20 $$\:{F}_{r}^{{\prime\:}}+{j}_{c}{w}_{c}^{{\prime\:}}={u}_{r}$$

21 $$\:{F}_{c}^{{\prime\:}}+{j}_{r}{w}_{r}^{{\prime\:}}={u}_{c}$$

Study might write this as,

22 $$\:{F}_{r}^{{\prime\:}}+{j}_{c}{w}_{c}^{{\prime\:}}={u}_{r}$$

23 $$\:{F}_{c}^{{\prime\:}}-\left({w}_{r}^{{\prime\:}}-{w}_{c}^{{\prime\:}}\right){j}_{r}-{j}_{r}{w}_{c}^{{\prime\:}}={u}_{c}$$

Combination formula works as,$\:\left({F}_{r}^{{\prime\:}}+i{F}_{c}^{{\prime\:}}\right)-i\left({j}_{r}+{ij}_{c}\right){w}_{c}^{{\prime\:}}+i\left({w}_{r}^{{\prime\:}}-{w}_{c}^{{\prime\:}}\right){j}_{r}=({u}_{r}+i{u}_{c})$(24).

To express the term $\:\left({w}_{c}^{{\prime\:}}-{w}_{r}^{{\prime\:}}\right){j}_{r}$as$\:{F}_{r}^{{\prime\:}}$, the equation becomes:

25 $$\:{F}_{r}^{{\prime\:}}+i\left({F}_{c}^{{\prime\:}}+{F}^{{\prime\:}}\right)-i\left({j}_{r}+{ij}_{c}\right){w}_{c}^{{\prime\:}}=\left({u}_{r}+{iu}_{c}\right)$$

It is stated as follows on the network side:

26 $$\:\left({F}_{r}^{{\prime\:}}+i\left({F}_{c}^{{\prime\:}}+{F}^{{\prime\:}}\right)\right){f}^{i\delta\:}-i\left({j}_{R}+{ij}_{C}\right){w}_{c}^{{\prime\:}}=({u}_{R}+{iu}_{C})$$

Where$\:\left({j}_{R}+{ij}_{C}\right)=\left({j}_{r}+{ij}_{C}\right){f}^{j\delta\:}$and$\:\left({u}_{R}+{iu}_{C}\right)=({u}_{r}+{iu}_{c}){f}^{i\delta\:}$. The following modification is made to this equation since it contains a term that depends on$\:\:\delta\:$.$\:{F}_{dummy}^{{\prime\:}}={F}^{{\prime\:}}*{f}^{i\delta\:}=\left({w}_{r}^{{\prime\:}}-{w}_{c}^{{\prime\:}}\right){j}_{r}{f}^{i\delta\:}$ == Steady state. The state equation for $\:{F}_{dummy}^{{\prime\:}}$ is as follows.

27 $$\:\frac{d}{dt}{F}_{dummy}^{{\prime\:}}=\frac{1}{{S}_{dummy}}\left[-{F}_{dummy}^{{\prime\:}}-i\left({w}_{r}^{{\prime\:}}-{w}_{c}^{{\prime\:}}\right){j}_{r}\right]$$

**Fig. 2:**
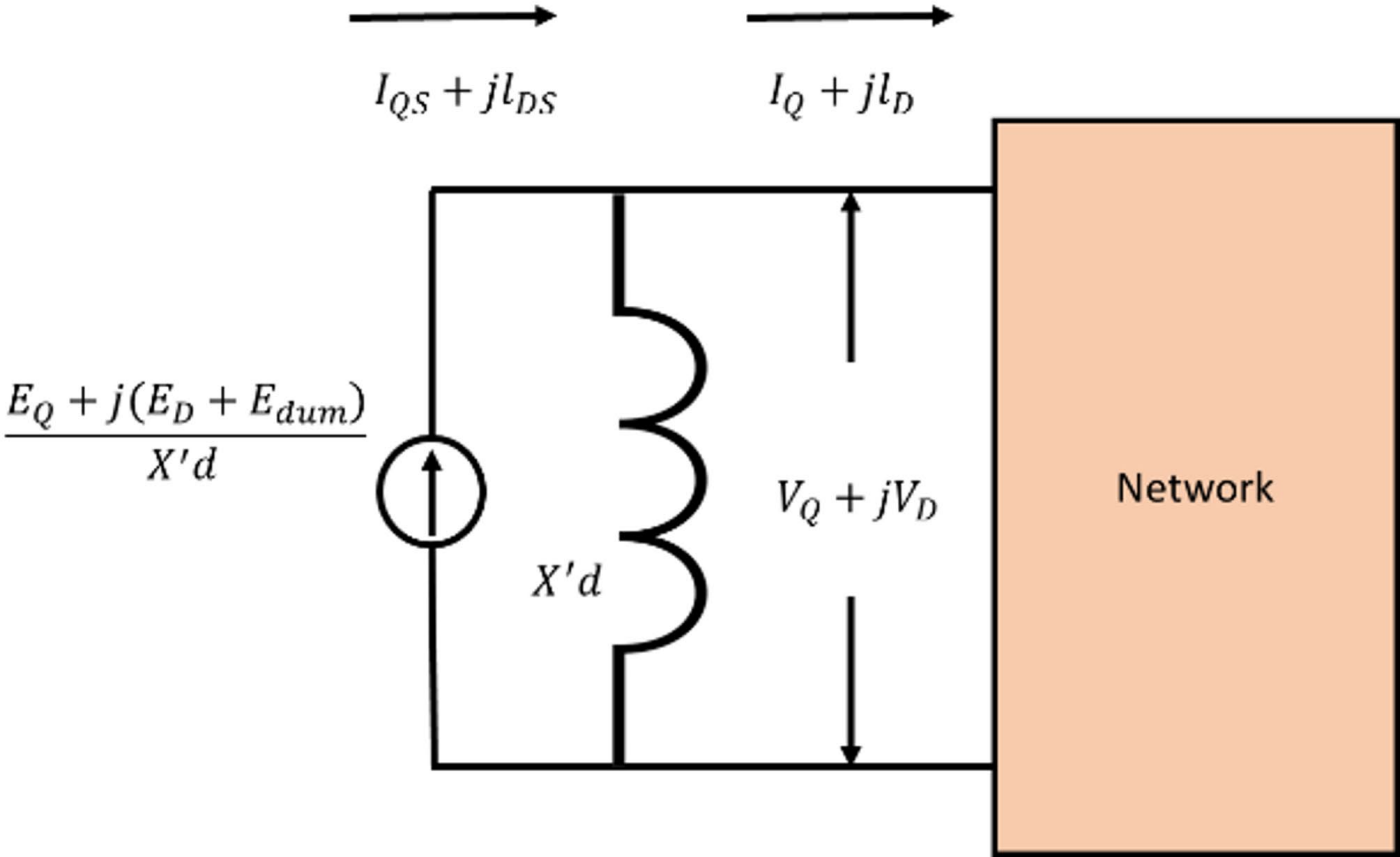
Representing Generators on the Network.

For the dummy coil, $\:{S}_{dummy}$ represents its open circuit time constant. In general, this is very low (~ 0.01). The models of the generators are continuous current sources with transient reactance parallel to the direct axis. The engine’s representation on the network’s side is shown in Fig. 2.

As a result, the solution to the final equation is:

28 $$\:{F}_{R}^{{\prime\:}}+i\left({F}_{C}^{{\prime\:}}+{F}_{dummy}\right)+i\left({j}_{R}+{ij}_{C}\right){w}_{c}^{{\prime\:}}=\left({u}_{R}+{iu}_{C}\right){w}_{c}^{{\prime\:}}=({u}_{R}+{iu}_{C})$$

Where, $$\:{F}_{R}^{{\prime\:}}+{iF}_{C}^{{\prime\:}}=({F}_{r}^{{\prime\:}}+{iF}_{c}^{{\prime\:}}){f}^{i\delta\:}$$

This approach approximates temporary saliency, but the level of resemblance can be modified by adjusting$\:{\:S}_{dummy}$. Using the aforesaid model, allows for the inclusion of $\:{w}_{c}^{{\prime\:}\:}$in the network. The total of the winds going into the shunted and the distribution system is the generator’s internal supply current.

29 $$\:{j}_{Ds}={j}_{C}+\frac{{u}_{R}}{{w}_{c}^{{\prime\:}}}$$

30 $$\:{j}_{Qs}={j}_{Q}+\frac{{u}_{C}}{{w}_{c}^{{\prime\:}}}$$

31 $$\:{j}_{r}+{ij}_{c}=({j}_{Qs}+{ij}_{Ds}){f}^{-i\delta\:}$$

### System description

**Fig. 3:**
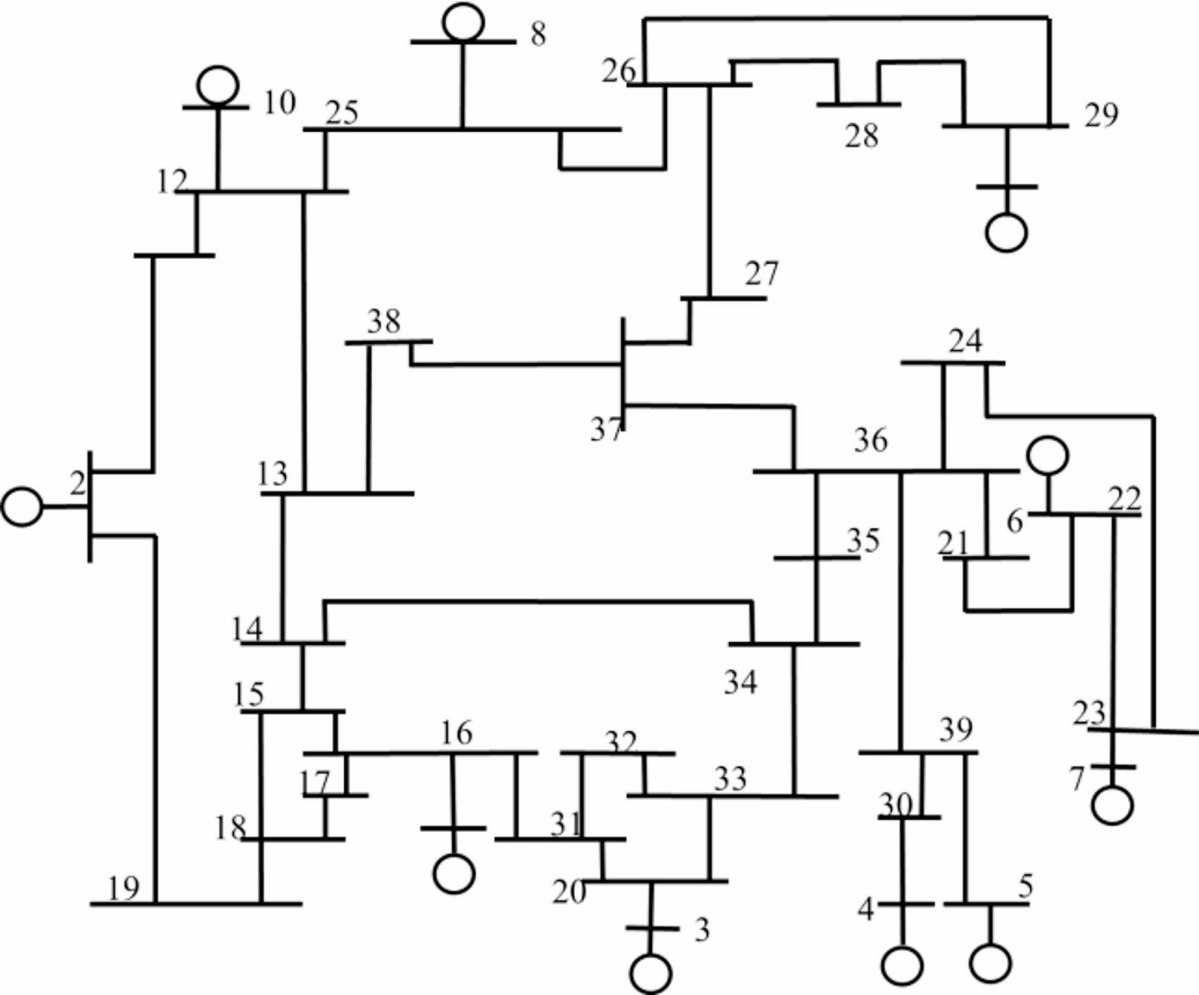
Diagram in one line for the 39 Bus Test System.

Bus No. 9 minor power units known as confined units and Bus No. 2 at the energy provider offer power to the commercial system under consideration. The 6th bus is thought to be slack. It is believed that the sector’s load is divided by import electrical supply through tie connections connecting Buses 2–19 with Buses 2–11 and stored power units. Twelve converters, 34 lines, and 39 buses make up the system. The system has a total electrical demand of 122.87 MW and an available power of 58.7MVAr. Energy from four distinct buses is supposed to be supplied by 4 DGs with a combined capacity of 50 MW. This work considers DG penetration of 22.6%. 39 Bus systems are displayed in Fig. 3.

Three steps are included in the analytical process. A 39-bus system is shown in scenarios − 1, 2, and 3, which also include four wind turbines, four solar cells, and two wind turbines and two solar cells for Large Low Load Generation (LLLG) faults, a transient stability assessment is conducted.

### Generator modeling

In this study, the synchronous generator is modeled using a standard simplified transient stability model. The analysis focuses on rotor dynamics, which determine whether the system remains stable after a disturbance.

The generator behavior is represented using the swing equation, which relates the mechanical input power and electrical output power to the rotor motion. The key parameters considered in this model include inertia constant, rotor angle, angular speed, and damping.

The electrical output power depends on the internal voltage, terminal voltage, and transfer reactance of the generator. Stator transients are neglected because they occur much faster and do not significantly affect transient stability analysis.

This simplified modeling approach is widely used in power system stability studies and ensures clarity and computational efficiency.

**System Configuration**.

The industrial power system considered in this work is a 39-bus network. It consists of multiple generators, transmission lines, and industrial loads.

The total system load is 122.87 MW and 58.7 MVAr. Bus 6 is considered as the slack bus. Power is supplied through both utility connections and captive generation units.

**DG Integration Strategy**.

The placement of solar and wind distributed generators (DGs) is carried out based on the following factors:

- Load concentration at different buses.
- Voltage profile of the system.
- Power flow conditions.

Three different scenarios are considered:

- Scenario 1: Only wind DGs.
- Scenario 2: Only solar DGs.
- Scenario 3: Hybrid combination of solar and wind DGs.

All DG units are connected through power electronic converters, which allow controlled injection of real and reactive power.

**Fault Modeling**.

To ensure consistency in analysis, the same type of fault is applied in all scenarios.

A three-phase symmetrical fault is considered, as it represents the most severe disturbance condition in a power system. The fault duration is fixed, and the clearing time is varied to determine the Critical Clearing Time (CCT).

## Result and analysis

The conditions necessary for integrating dispersed solar and wind generators into industrial power systems are presented in this paper. Therefore, for the best results, a Windows-10 platform and MATLAB-R2024a/Simulink must be used for the transient stability analysis and simulations. To model power systems, simulate the system, and analyze how the incorporation of RES influences transient stability, several conditions must be fulfilled. We present the changes in voltage magnitudes, angles, as well as power flows before and after the connection of DGs in both scenarios. The values of CCT, real power, relative power, and active power for each of the three scenarios are shown in Table 1. The values presented in Table 1 represent the total system active and reactive power under fault conditions after DG integration. These values correspond to the system response during transient conditions and not the steady-state load demand.

**Table 1:** Bus systems (39) with DGs.

Scenarios	Real Power (MW)	Fault Evaluated	Critical Clearing Time (CCT)	Active Power (MW)	Reactive Power (MVAr)
**(1) 4- Wind Turbine**	3.8	3-phase	0.024848 s	200.1889	69.2241
**(2) 4- Solar Cells**	25	3-phase	0.31687 s	1165	583
**(3) 2- Wind Turbine and 2- Solar Cell**	38	line-to-line	0.029798 s	740	776

### Impact of hybrid DGs on transient stability: an analysis of power flow and CCT

This transient stability analysis calculates stability margins before and after placing Hybrid DGs, comparing them to a stop time to determine a bus’s stability. A stable bus has a margin greater than or equal to the stop time, while unstable generators transition from stable to unstable after DG placement. Stability margins determine a system’s stability, while CCT is the time within which a fault must be cleared to maintain stability. The stop time is a threshold value used to determine system stability. The code calculates and displays stability margins for each bus, identifying generators that transition from stable to unstable, allowing assessment of the impact of DG placement on system stability.

For example, the increase in CCT for faults at buses 11 and 12 is not significant. However, at bus 21, the CCT increases considerably after placing DGs due to the reversal of active power flow between buses 36 and 21. Similarly, at bus 35, the CCT increases significantly because of active power generation at bus 36. In terms of power flows, there is a decrease in active power flow from bus 11 to 12 and bus 10 to 12, but an increase in reactive power flow from bus 11 to 12. These changes in power flow directly affect the CCT, Table 2 indicating that significant changes in power flow due to DG placement can lead to substantial changes in the system’s CCT.

**Table 2:** Impact of DG Placement on Power Flows and CCT.

From Bus	To Bus	Real Power Flow (MW)	Reactive Power Flow (MVAr)
11	12	Decreased	Increased
10	12	Decreased	-
36	21	Reversed	-

The placement of DGs impacts the system’s stability by altering power flows, which in turn affects the CCT. Buses with significant changes in power flow experience notable changes in CCT, highlighting a direct relationship between power flow dynamics and system stability.

### Scenario − 1(4- wind turbine)

**Fig. 4:**
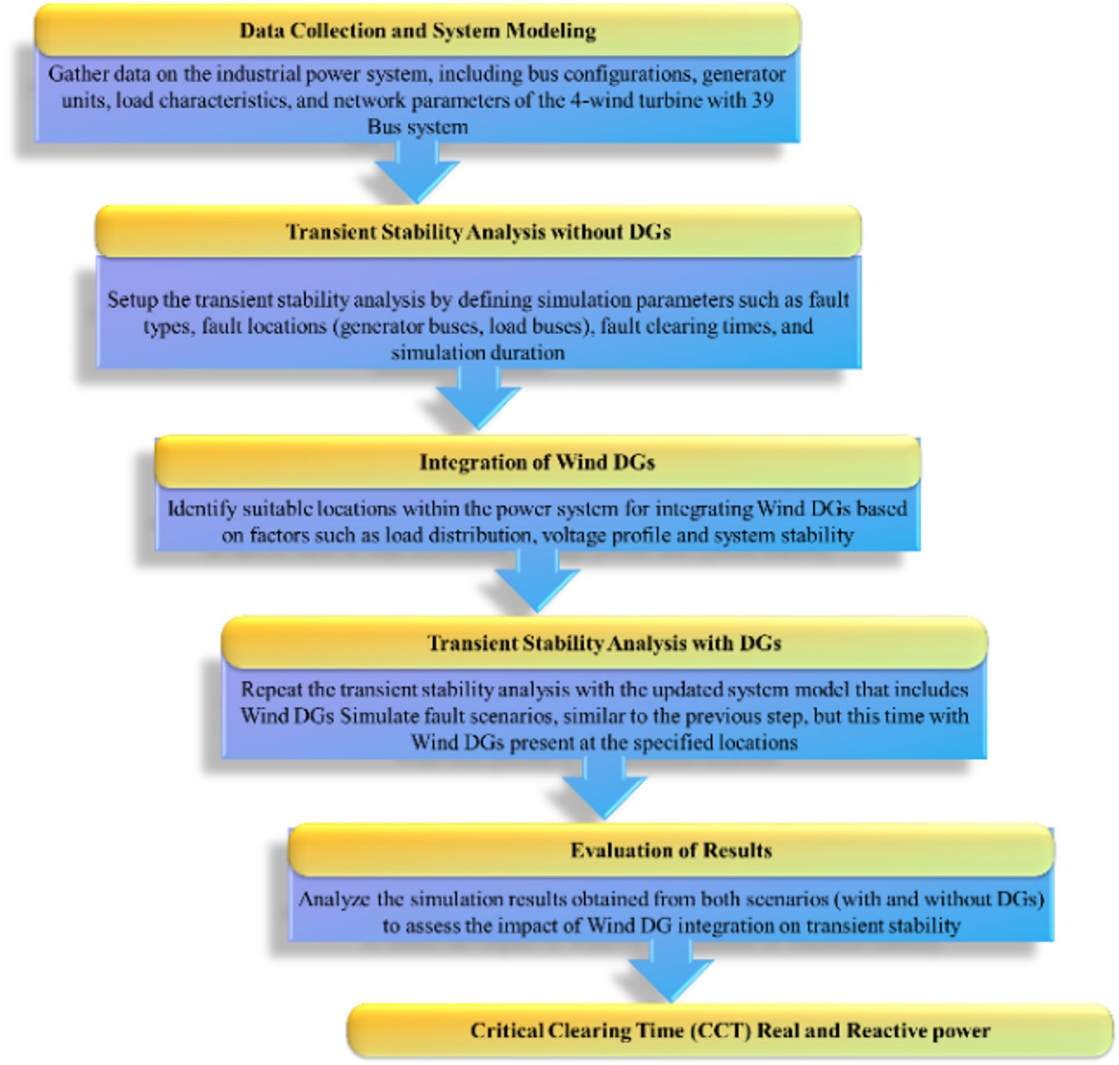
The configuration of Scenario − 1.

**Fig. 5:**
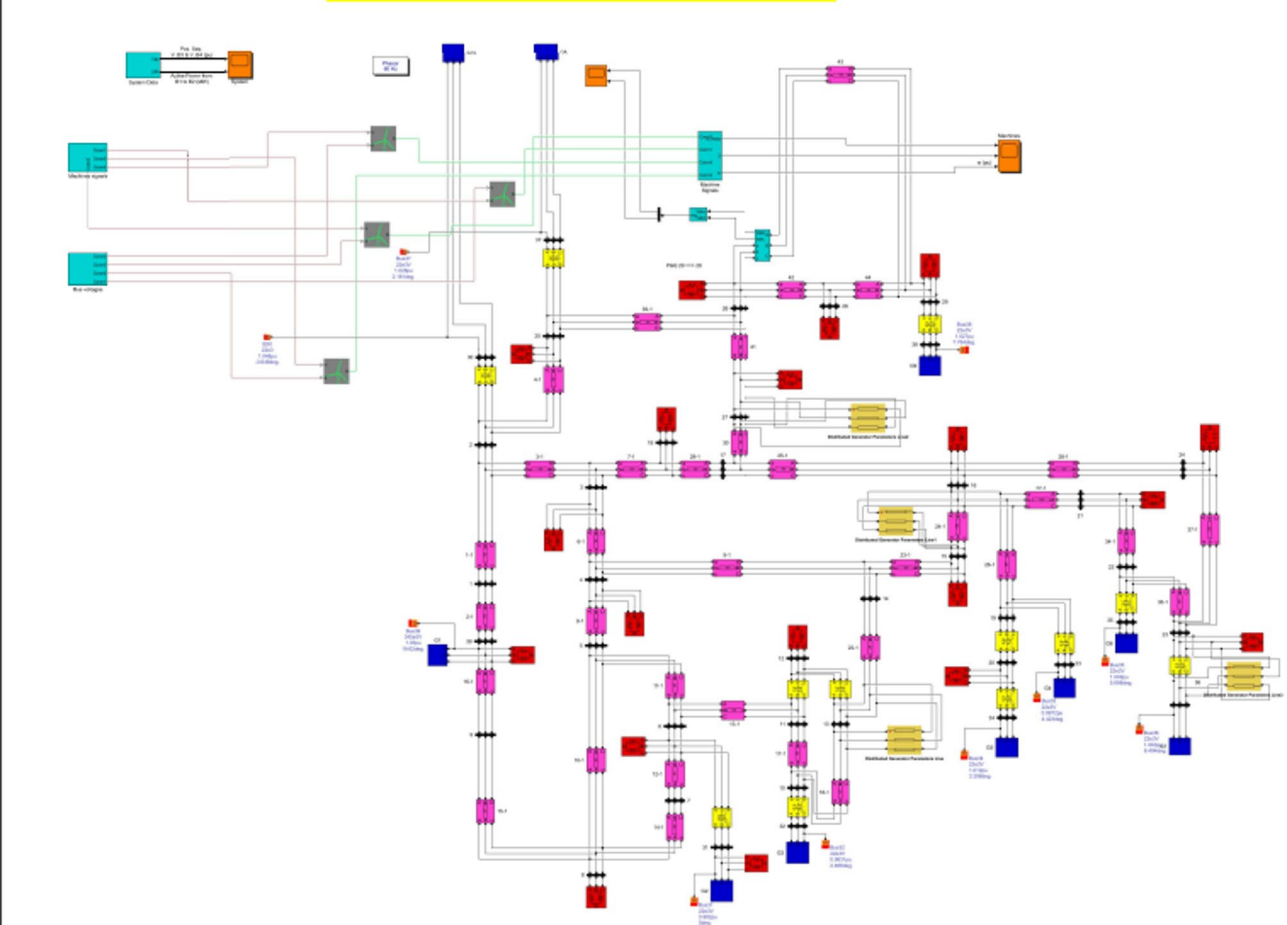
wind turbine (4) with 39 Bus systems (Scenario − 1).

**Fig. 6:**
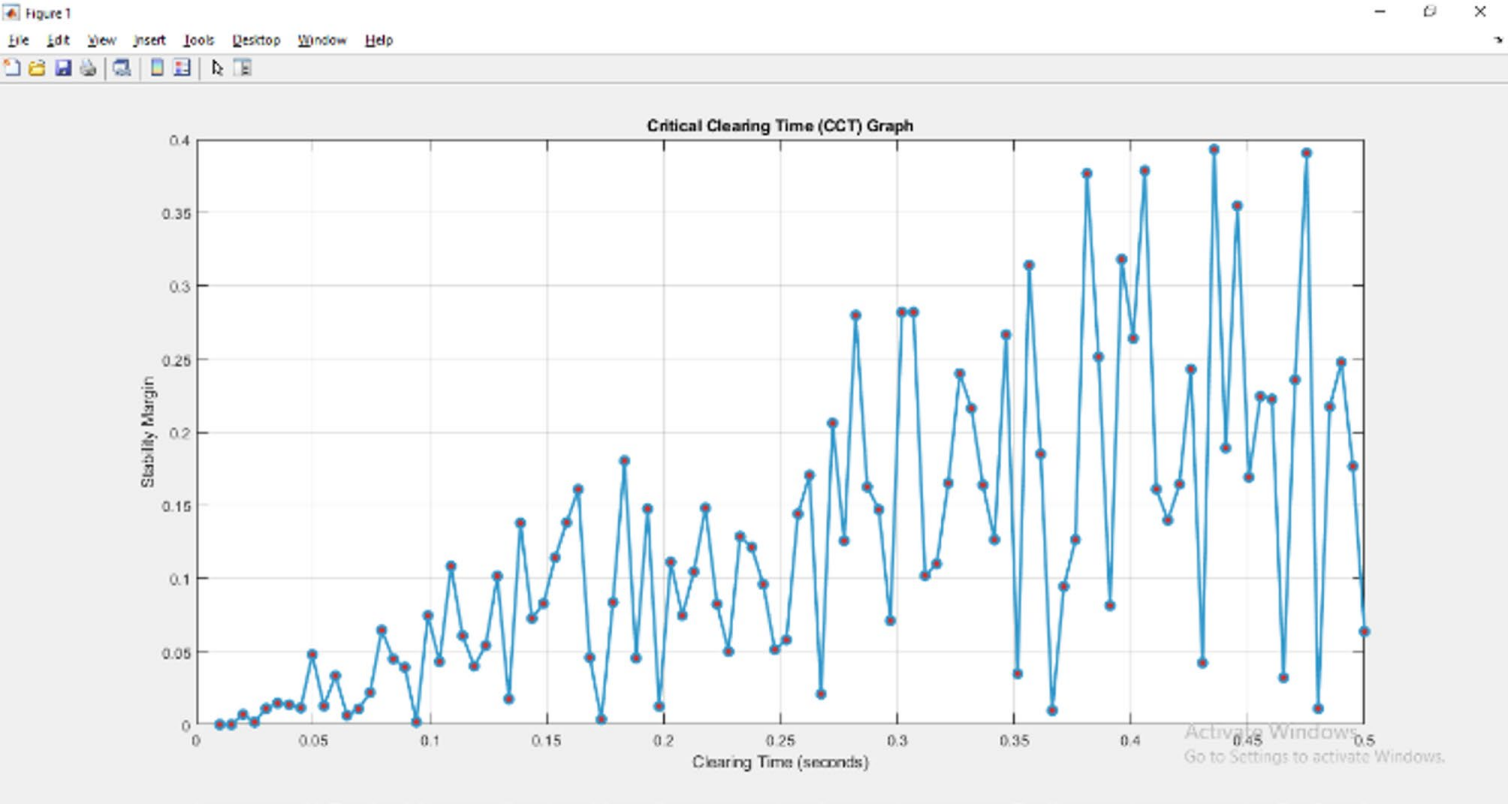
CCT of a 4 wind turbine in scenario-1.

**Fig. 7:**
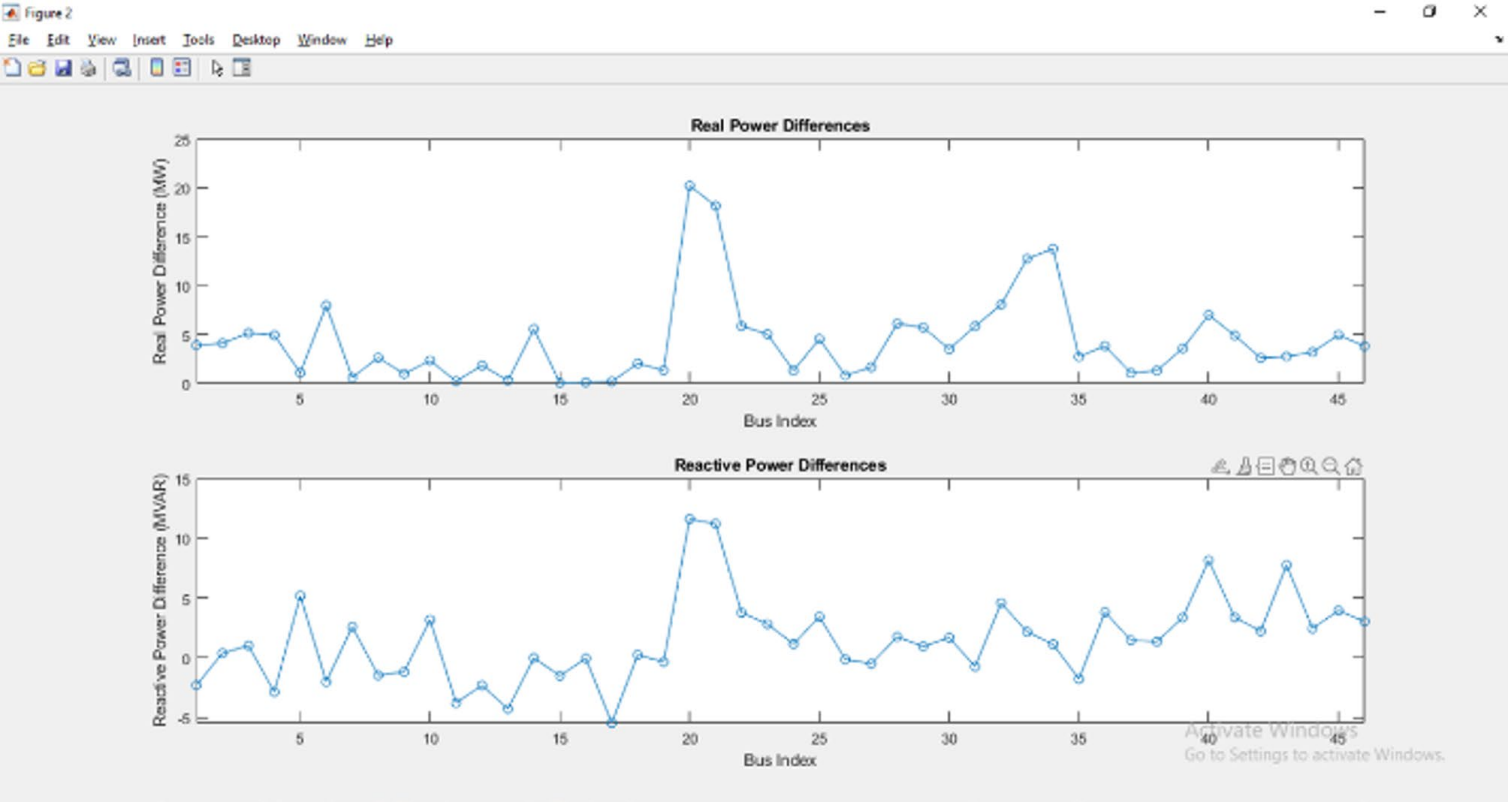
Scenario − 1 Active and Reactive Power Differences.

**Fig. 8:**
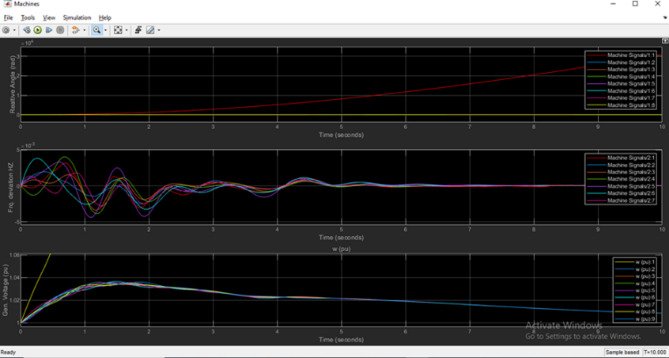
Dynamic Response at Bus No. 2.

The structure of scenario-1 is shown in Figs. 4 and 5 depicts scenario-1, 4 wind turbine with a 39-bus system. In this analysis of the transient stability, a conventional generator is connected to bus 1 with 100 MW rated output and an inertial constant of 3.5. Furthermore, a Scenario – 1 involves 4 wind DGs located at buses 2,5,10 and 20, with their capacities of 50 MW, 30 MW, 40 MW, and 60 MW, an inertia constant of 1.0, and additionally, there is a three-phase fault with buses 13, 15, 27, and 36. The system leaves a sustainability horizon line at 0.4 and a voltage threshold of 0.8 volts are mandatory, to investigate the features of stability of the power system at any conditions. These values are useful in assessing abilities of the system to counter any interference and in guaranteeing a stable power supply. As seen in Fig. 6, for scenario 1, the CCT is approximately 0.0248485 s. Figure 7 depicts the active (200.1889 MW) and reactive (69.2241 MVAr) power differences for scenario (1) Before installing DGs, Fig. 8 displays the currents at the generator buses, the rate of deviation, and the associated angle in radians for the fault at Bus No. (2) The fault clearing time ($\:{T}_{clear}$​) is 0.1 s, indicating the case study parameters.

### Scenario − 2 (4- Solar cell)

**Fig. 9:**
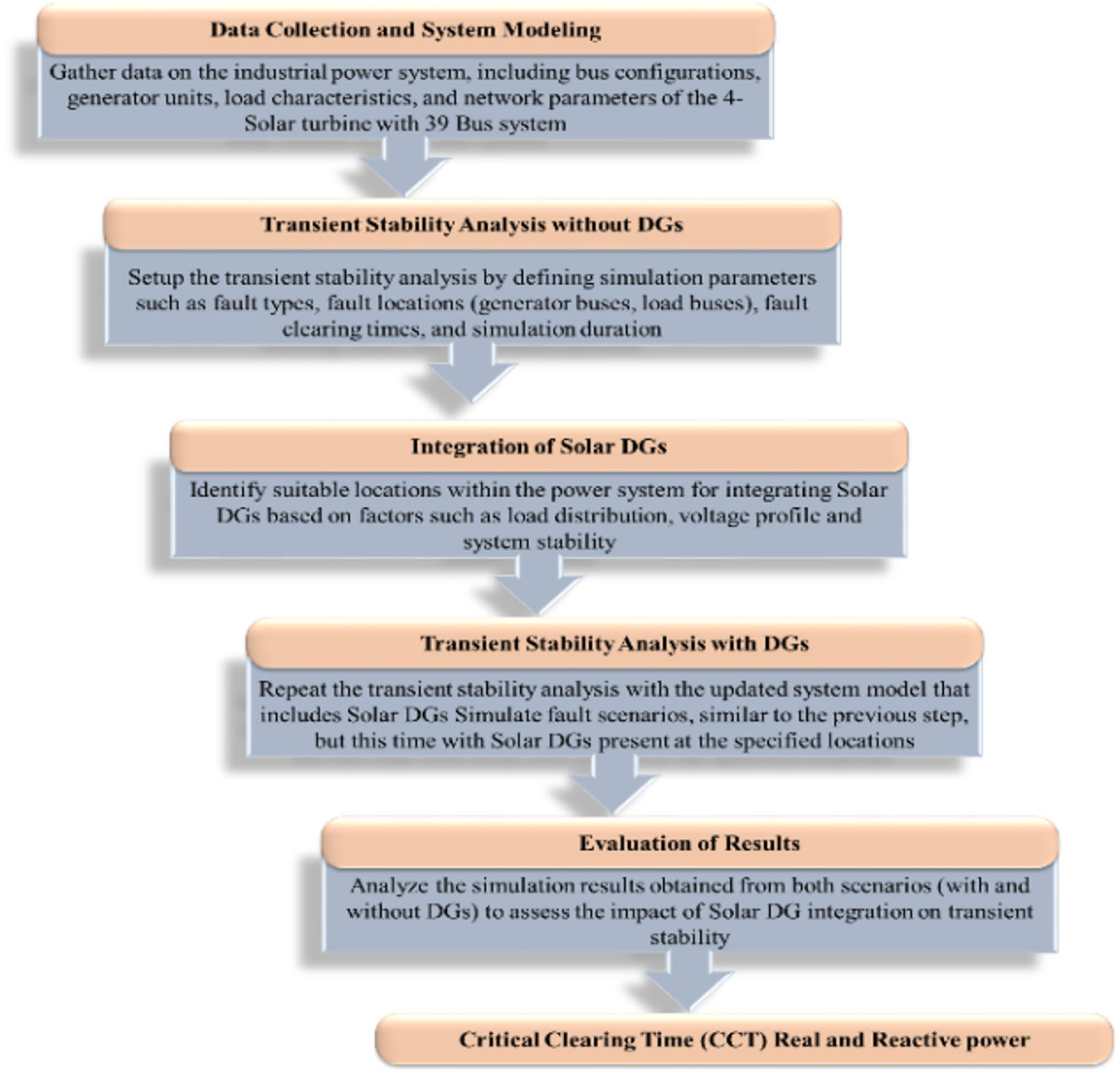
The configuration of Scenario − 2.

**Fig. 10:**
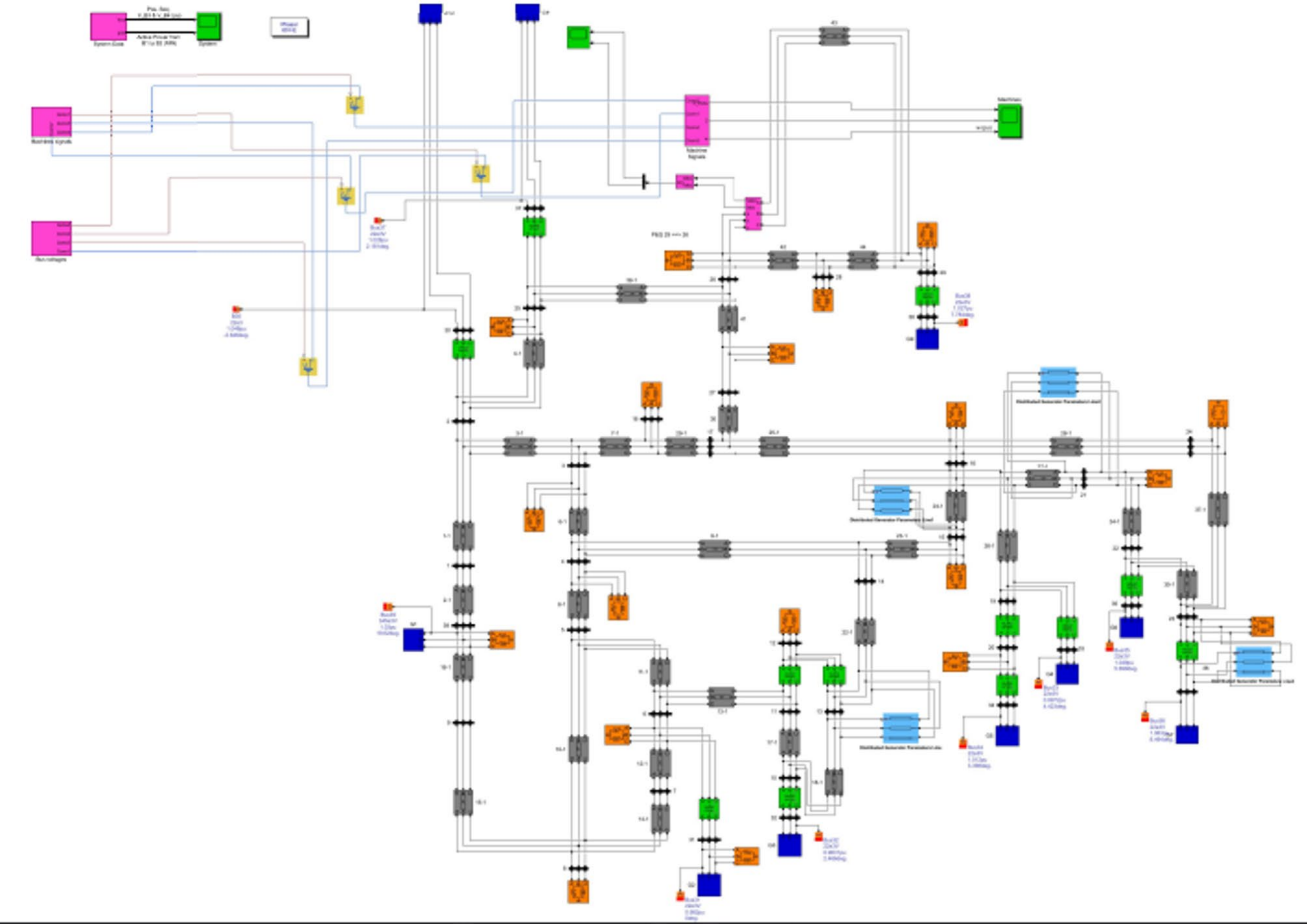
Solar cell (4) with 39 Bus systems (Scenario − 2).

**Fig. 11 C:**
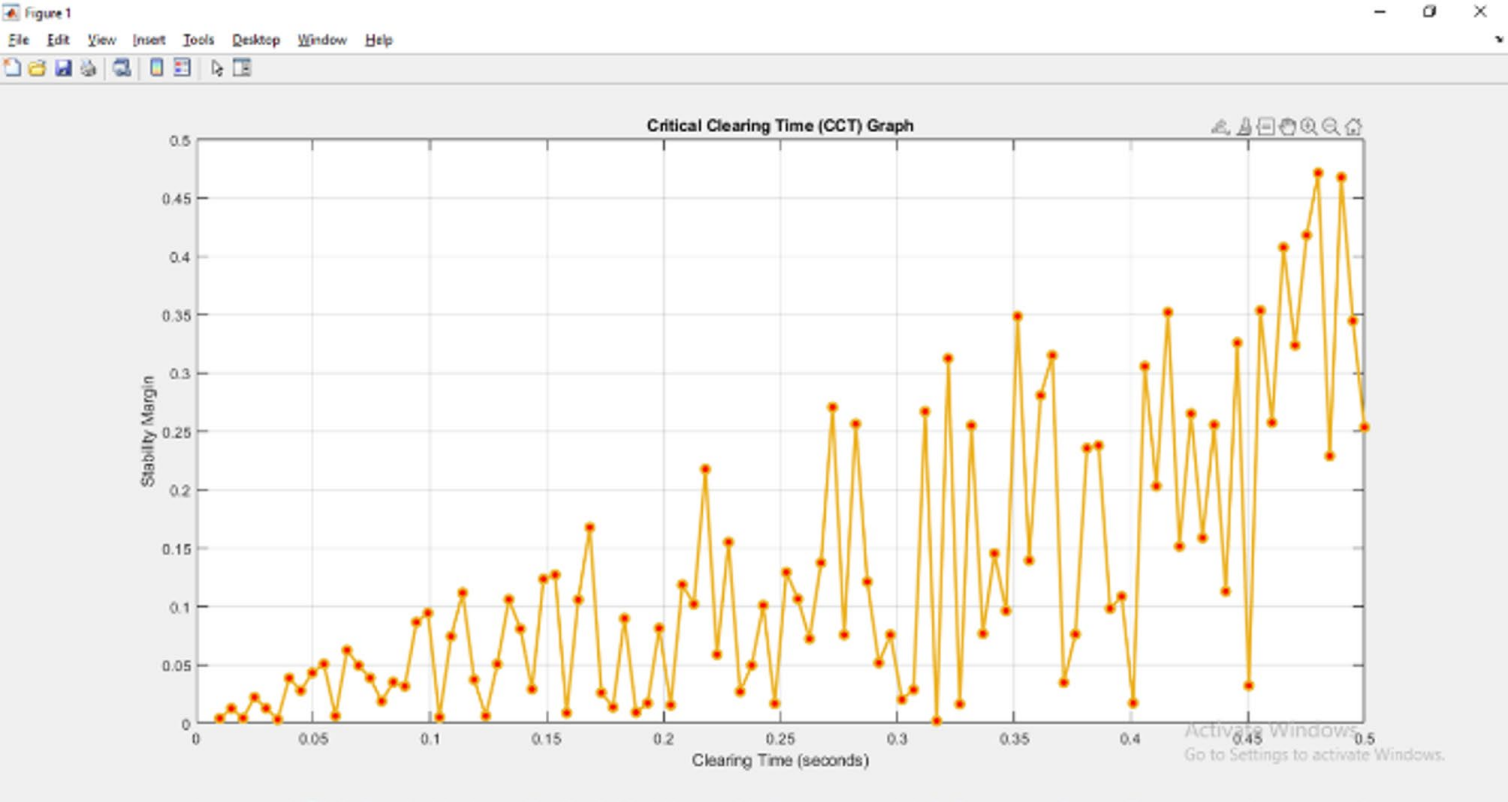
CT of a 4 Solar cell in scenario-2.

**Fig. 12:**
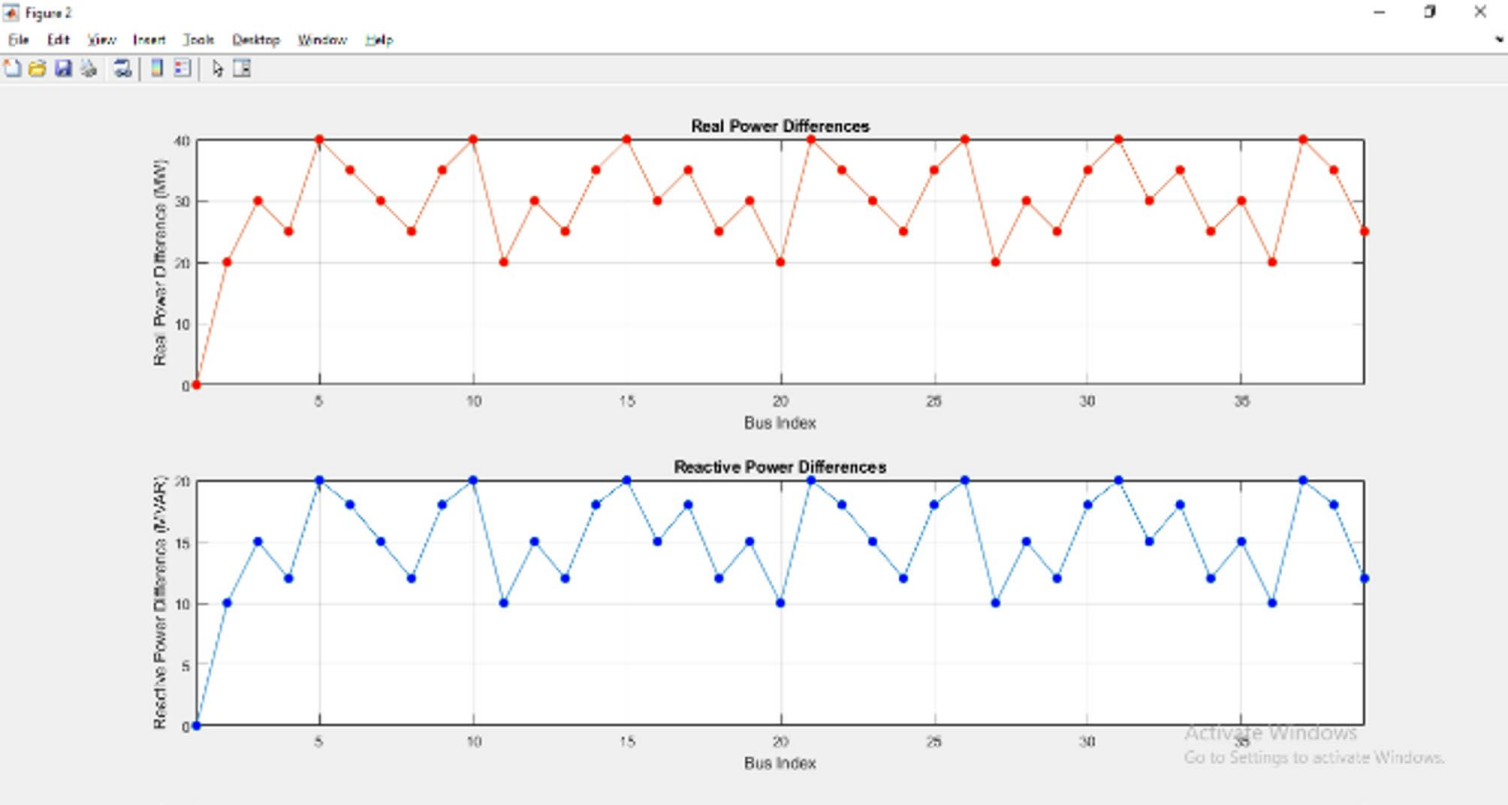
Scenario − 2 Active and Reactive Power Differences.

**Fig. 13:**
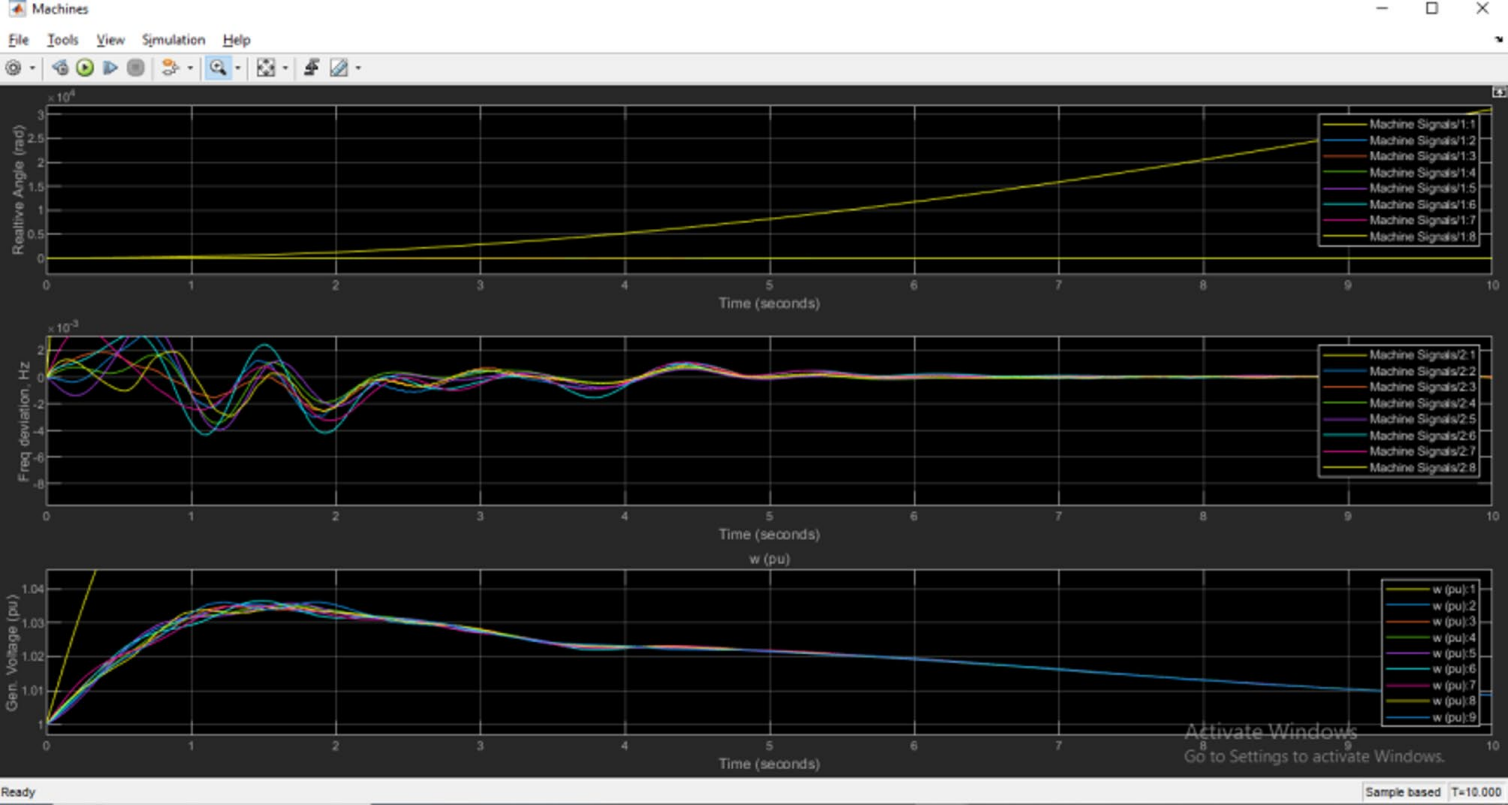
Dynamic Response at Bus No. 2.

Figure 9 depicts the setup for scenario 2. A 39-bus system with four solar cells is shown in scenario-2 in Fig. 10. For Buses Nos. 21, 13, 31, and 15, four solar cell power DGs are being evaluated. An inertial constant of 3.5 and a conventional generator with a 100 MW rated output are coupled to bus 1 in this transient stability examination. Additionally, Scenario-2 involves 4 solar DGs located at buses 13, 15, 21, and 31, with their capacities of 30 MW, 40 MW, 50 MW, and 60 MW, a 1.0 inertia constant, and a 3-phase fault type is integrated into the system. A voltage threshold of 0.8 volts is required in order to explore the characteristics of power system stability under all circumstances. The system exits a sustainability horizon line at 0.4. These figures are helpful in determining how well the system can withstand interference and provide a steady supply of electricity. The Scenario − 1 CCT takes around 0.31687 s, as shown in Fig. 11. The power disparities for Scenario − 2 Active (1165 MW) and Reactive (583 MVAR) are shown in Fig. 12. Figure 13 shows the currents at the generator buses for the problem at Bus No. 2, along with the corresponding angle in radians and the rate of deviation. fault clearing time ($\:{T}_{clear}$) is 0.1 s, indicating that the case study.

### Scenario − 3 (2- wind turbine and 2- Solar Cell)

**Fig. 14:**
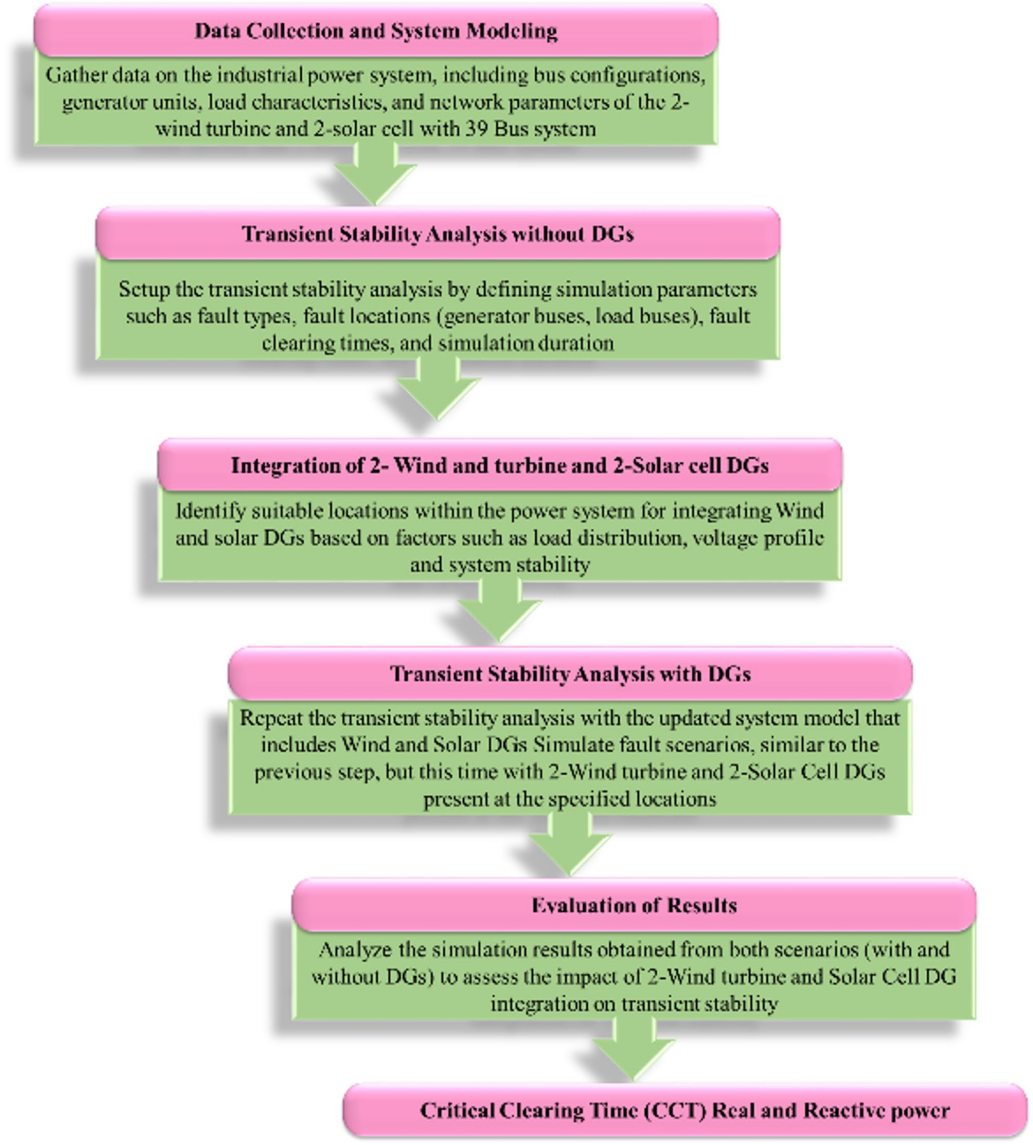
The configuration of Scenario − 3.

**Fig. 15:**
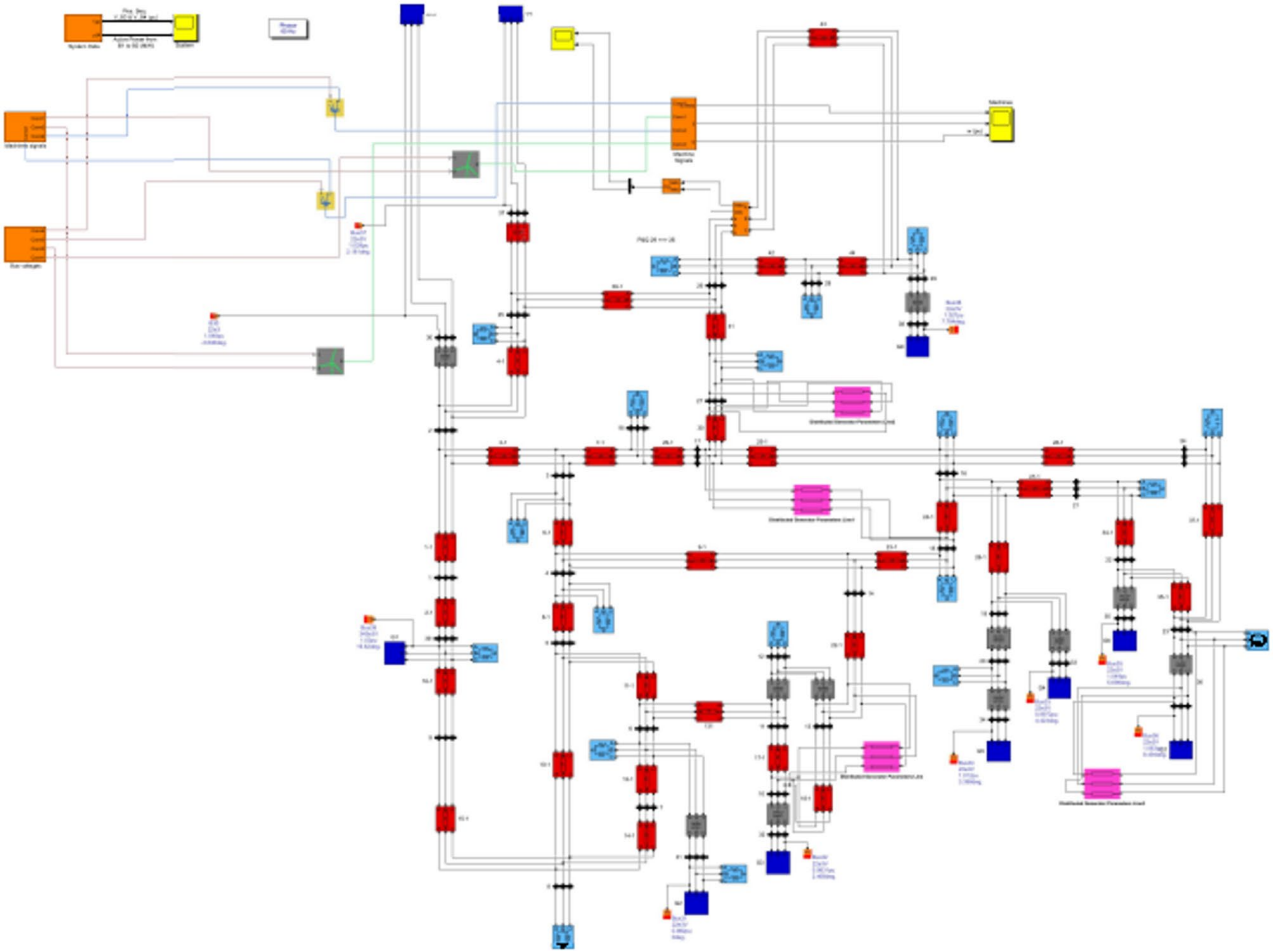
Wind turbine (2) and Solar Cell (2) with 39 Bus systems (Scenario − 3).

**Fig. 16:**
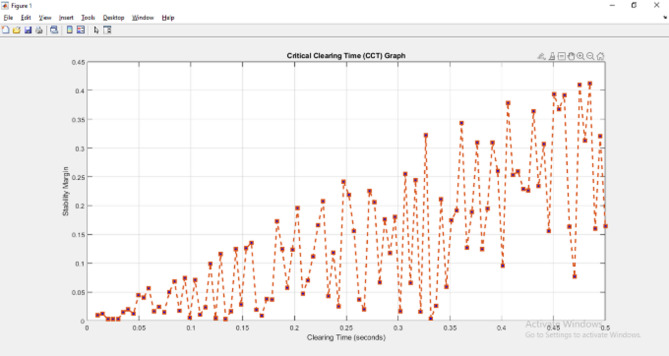
CCT of a wind turbine (2) and Solar Cell (2) in scenario-3.

**Fig. 17:**
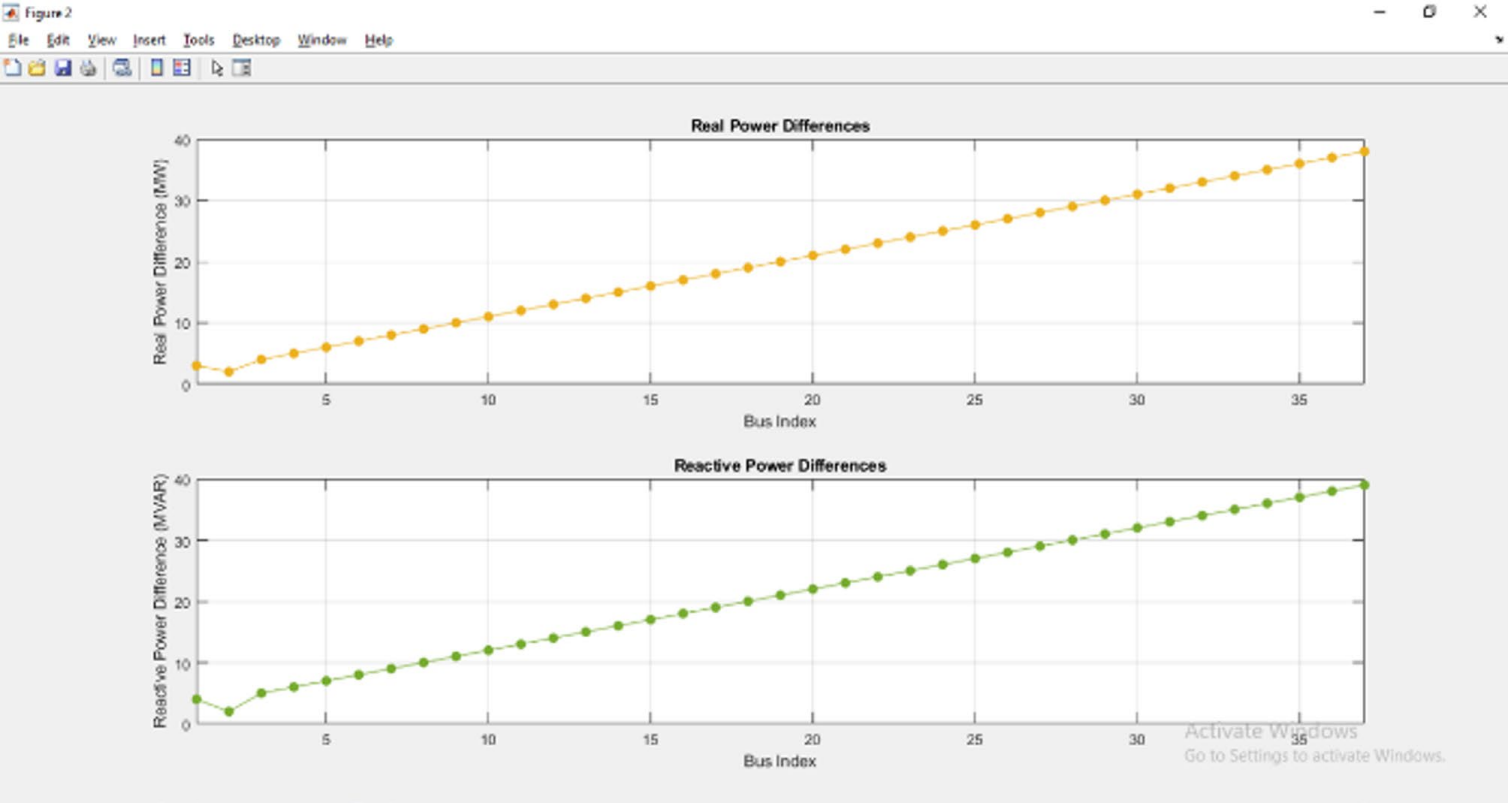
Scenario − 1 Active and Reactive Power Differences.

**Fig. 18:**
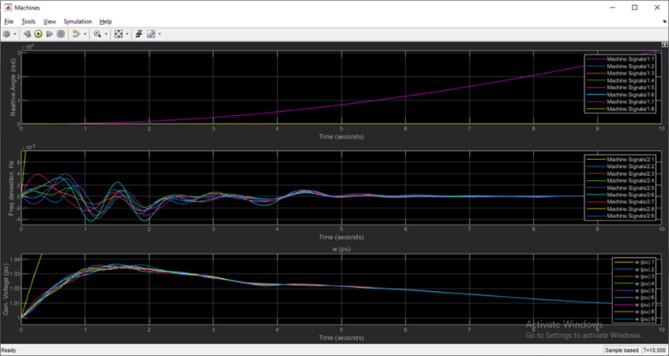
Dynamic Response at Bus No. 2.

Figure 14 depicts the setup for scenario 3. Using a 39-bus system, Fig. 15 shows scenario − 3, two wind turbines, and two solar cells. For buses nos. 10, 17, 5, and 20, 2 -wind and 2- solar cells power DGs are under consideration. An inertial constant of 3. 5 and a conventional generator with a 100 MW rated output are coupled to bus 1 in this transient stability investigation. Scenario − 3 CCT takes around 0.029798 s, as shown in Fig. 16. It includes solar DGs at buses 10 and 17, with capacities if 30 MW and 40 MW and wind DGs is located at buses 5 and 20, with capacities of 60 MW and 70 MW respectively, a 1.0 inertia constant, and a line-to-line phase fault type is incorporated into the system. Figure 17 displays the power differences for Scenario − 3 Active (740 MW) and Reactive (776 MVAr). Figure 18 shows the currents at the generator buses for the problem at Bus No. 2, along with the corresponding angle in radians and the rate of deviation. $\:{T}_{clear}$is 0.10 s, indicating that the case study. A voltage threshold of 0.8 volts is required to examine the stability aspects of the power system under all circumstances. The system leaves a sustainability horizon line at 0. 4. The system’s ability to withstand interferences and ensure a steady power supply may be evaluated using these metrics.

### Impact of DG integration on stability

The results show that the integration of distributed generators improves the transient stability of the system.

Among the three scenarios, solar DG provides the highest improvement in stability, as indicated by the maximum Critical Clearing Time. This is because solar systems, connected through power electronic converters, can provide better voltage support during disturbances.

Wind DGs show moderate improvement due to their dependence on mechanical dynamics and limited reactive power support.

The hybrid configuration provides intermediate results. This indicates that combining different DG types introduces complex interactions, which may not always lead to maximum stability improvement.

When compared to the baseline case, all DG configurations improve system stability, but the level of improvement depends on the type and placement of DG.

## Conclusion

This paper studied the impact of solar and wind distributed generators on the transient stability of a 39-bus industrial power system. A baseline case and three DG scenarios were analyzed using Critical Clearing Time as the main performance metric. The results show that DG integration improves system stability, with solar DG providing the highest improvement. Wind DG shows moderate performance, while hybrid DG shows mixed results due to interaction effects. The study highlights the importance of proper DG placement and system design in improving stability. Future work will focus on real-time validation and advanced control strategies.While DG integration improves system stability, it also involves economic and technical trade-offs. Solar DG systems require inverter infrastructure, while wind DG systems depend on mechanical components and site conditions.

The selection of DG type must consider both stability performance and cost implications to ensure practical feasibility.

### Limitation & future scope

The dependence on simulation-based analysis, which could not adequately portray the intricacies and changes of the actual world, is one of the work’s limitations. Empirical data and field testing are thus necessary for validation. Future studies might examine advanced control approaches to further improve transient stability in industrial power systems, evaluate findings through field experiments, and investigate the practical application of the suggested integration solutions.

## Data Availability

The datasets used and/or analysed during the current study available from the corresponding author on reasonable request.
